# Concept-based query expansion for retrieving gene related publications from MEDLINE

**DOI:** 10.1186/1471-2105-11-212

**Published:** 2010-04-28

**Authors:** Sérgio Matos, Joel P Arrais, João Maia-Rodrigues, José Luis Oliveira

**Affiliations:** 1 Institute of Electronics and Telematics Engineering of Aveiro (IEETA), University of Aveiro, 3810-193 Aveiro, Portugal; 2 Computational Structural Biology, Department of Structural Biology, Stanford University School of Medicine, Stanford CA 94305, USA

## Abstract

**Background:**

Advances in biotechnology and in high-throughput methods for gene analysis have contributed to an exponential increase in the number of scientific publications in these fields of study. While much of the data and results described in these articles are entered and annotated in the various existing biomedical databases, the scientific literature is still the major source of information. There is, therefore, a growing need for text mining and information retrieval tools to help researchers find the relevant articles for their study. To tackle this, several tools have been proposed to provide alternative solutions for specific user requests.

**Results:**

This paper presents QuExT, a new PubMed-based document retrieval and prioritization tool that, from a given list of genes, searches for the most relevant results from the literature. QuExT follows a concept-oriented query expansion methodology to find documents containing concepts related to the genes in the user input, such as protein and pathway names. The retrieved documents are ranked according to user-definable weights assigned to each concept class. By changing these weights, users can modify the ranking of the results in order to focus on documents dealing with a specific concept. The method's performance was evaluated using data from the 2004 TREC genomics track, producing a mean average precision of 0.425, with an average of 4.8 and 31.3 relevant documents within the top 10 and 100 retrieved abstracts, respectively.

**Conclusions:**

QuExT implements a concept-based query expansion scheme that leverages gene-related information available on a variety of biological resources. The main advantage of the system is to give the user control over the ranking of the results by means of a simple weighting scheme. Using this approach, researchers can effortlessly explore the literature regarding a group of genes and focus on the different aspects relating to these genes.

## Background

Advances in biotechnology, together with the widespread use of high-throughput methods for gene analysis, have helped shifting the focus of biological research from specific genes and proteins to a more systemic analysis of the underlying biological problem. Researchers now face the increasing need to plan their experiments and analyse the resulting datasets in view of the quickly expanding biomedical information available [1,2]. Although much of this knowledge is being annotated in the various existing biomedical databases, keeping these up-to-date is a difficult task due to the rapid emergence of new results and the exponential increase of published articles. Therefore, many relevant research outcomes are still enclosed as free-text in the literature, which remains the major source of information for researchers [3]. A significant challenge for researchers is how to identify the most relevant articles for their specific study, and how to integrate this with the scientific knowledge annotated in biological databases, in an efficient manner [4]. This integrated view of the literature, in the framework of a more systematized and formalized knowledge extracted from databases and ontologies, and the ability to analyse large datasets (a list of genes from a microarray experiment, for example), are two important requisites for biological data analysis [2].

The most popular biomedical information retrieval system, PubMed, gives researchers access to over 17 million citations from a broad collection of scientific journals, indexed by the MEDLINE literature database. PubMed facilitates access to the biomedical literature by combining the Medical Subject Headings (MeSH) based indexing from MEDLINE, with Boolean and vector space models for document retrieval, offering a single interface from which these journals can be searched [5]. However, and despite these strong points, there are some limitations in using PubMed or other similar tools. A first limitation comes from the fact that keyword-based searches usually lead to underspecified queries, which is a main problem in any information retrieval (IR) system [6]. This usually means that users will have to perform various iterations and modifications to their queries in order to satisfy their information needs. This process is well described in [7] in the context of information-seeking behaviour patterns in biomedical information retrieval. Another drawback is that PubMed does not sort the retrieved documents in terms of how relevant they are for the user query. Instead, the documents satisfying the query are retrieved and presented in reverse date order. This approach is suitable for such cases in which the user is familiar with a particular field and wants to find the most recent publications. However, if the user is looking for articles associated with several query terms and possibly describing relations between those terms, the most relevant documents may appear too far down the result list to be easily retrieved by the user.

To address the issues mentioned above, several tools have been developed in the past years that combine information extraction, text mining and natural language processing techniques to help retrieve relevant articles from the biomedical literature [8]. Most of these tools are based on the MEDLINE literature database and take advantage of the domain knowledge available in databases and resources like the Entrez Gene, UniProt, GO or UMLS to process the titles and abstracts of texts and present the extracted information in different forms: relevant sentences describing a biological process or linking two or more biological entities, networks of interrelations, or in terms of co-occurrence statistics between domain terms. One such example is the GoPubMed tool [9], which retrieves MEDLINE abstracts and categorizes them according to the Gene Ontology (GO) and MeSH terms. Another tool, iHOP [10], uses genes and proteins as links between sentences, allowing the navigation through sentences and abstracts. The AliBaba system [11] uses pattern matching and co-occurrence statistics to find associations between biological entities such as genes, proteins or diseases identified in MEDLINE abstracts, and presents the search results in the form of a graph. EBIMed [12] finds protein/gene names, GO annotations, drugs and species in PubMed abstracts showing the results in a table with links to the sentences and abstracts that support the corresponding associations. FACTA [13] retrieves abstracts from PubMed and identifies biomedical concepts (e.g. genes/proteins, diseases, enzymes and chemical compounds) co-occurring with the terms in the user's query. The concepts are presented to the user in a tabular format and are ranked based on the co-occurrence statistics or on pointwise mutual information. More recently, there has been some focus on applying more detailed linguistic processing in order to improve information retrieval and extraction. Chilibot [14] retrieves sentences from MEDLINE abstracts relating to a pair (or a list) of proteins, genes, or keywords, and applies shallow parsing to classify these sentences as interactive, non-interactive or simple abstract co-occurrence. The identified relationships between entities or keywords are then displayed as a graph. Another tool, MEDIE [15], uses a deep-parser and a term recognizer to index abstracts based on pre-computed semantic annotations, allowing for real-time retrieval of sentences containing biological concepts that are related to the user query terms.

Despite the availability of several specific tools, such as the ones presented above, we feel that the demand for finding references relevant for a large set of is still not fully addressed. This constitutes an important query type, as it is a typical outcome of many experimental techniques. An example is a gene expression study, in which, after measuring the relative mRNA expression levels of thousands of genes, one usually obtains a subset of differentially expressed genes that are then considered for further analysis [16,17]. The ability to rapidly identify the literature describing relations between these differentially expressed genes is crucial for the success of data analysis. In such cases, the problem of obtaining the documents which are more relevant for the user becomes even more critical because of the large number of genes being studied, the high degree of synonymy and term variability, and the ambiguity in gene names.

While it is possible to perform a composite query in PubMed, or use a list of genes as input to some of the IR tools described above, these systems do not offer a retrieval and ranking strategy which ensures that the obtained results are sorted according to the relevance for the entire input list. A tool more oriented to analysing a set of genes is microGENIE [18], which accepts a set of genes as input and combines information from the UniGene and SwissProt databases to create an expanded query string that is submitted to PubMed. A more recently proposed tool, GeneE [19], follows a similar approach. In this tool, gene names in the user input are expanded to include known synonyms, which are obtained from four reference databases and filtered to eliminate ambiguous terms. The expanded query can then be submitted to different search engines, including PubMed. In this paper, we propose QuExT (Query Expansion Tool), a document indexing and retrieval application that obtains, from the MEDLINE database, a ranked list of publications that are most significant to a particular set of genes. Document retrieval and ranking are based on a concept-based methodology that broadens the resulting set of documents to include documents focusing on these gene-related concepts. Each gene in the input list is expanded to its various synonyms and to a network of biologically associated terms, namely proteins, metabolic pathways and diseases. Furthermore, the retrieved documents are ranked according to user-defined weights for each of these concept classes. By simply changing these weights, users can alter the order of the documents, allowing them to obtain for example, documents that are more focused on the metabolic pathways in which the initial genes are involved.

## Implementation

The main idea behind QuExT was to create a document indexing and retrieval system combining three main advantages: a) allow the study of a set of biological entities, namely a set of genes, in a single query; b) allow expanding the search queries using co-related terms; and c) allow the user to change the order of the retrieved documents through a simple weighting scheme. In order to achieve this, we developed a query expansion strategy based on networks of biological concepts related to the genes. These networks were created by integrating information from various biological databases and ontologies, as originally proposed in [20]. The query terms are expanded using these concepts, and document ranking is achieved by assigning weights to these concepts. With this method, we aim to address many problems related to genomic information retrieval, namely: how to deal with the various names and symbols used for a particular gene [21] and how to account for this variability when matching documents to the user query [22-24]; how to address the user information need in terms of what are the concepts related to the genes that the user is most interested in; what is the best query expansion strategy for this specific problem; and which are the best terms to use for expanding the query [25]. The application development was therefore focused on four main aspects:

1. Gene name mapping and normalization, to deal with the different identifiers used in different databases;

2. Query expansion, to deal with the high degree of synonymy in gene names and also to obtain documents that not only refer to the genes but also to related concepts;

3. Retrieval and ranking of the results, so that the top documents are the most relevant to the entire set or subsets of the input genes;

4. Term re-weighting, which allows users to change the influence of the different concept types in document ranking, therefore focusing on specific aspects related to the gene list.

**Figure 1:**
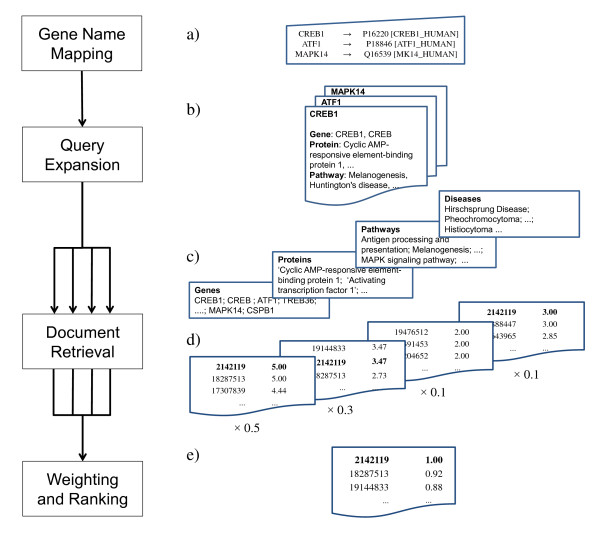
**QuExT query expansion procedure**. Query expansion and document ranking procedure: a) gene names in the query are converted to internal identifiers; b) the different gene name synonyms and related concepts for each gene are used to expand the user query; c) the index is searched for each term in the expanded query; d) resulting documents and scores are kept in separate lists (one for each concept class); e) results are assembled using the user-defined weights for each concept and the final results are normalized in relation to the highest score.

Each of these stages is described in detail in the next sections. The complete procedure is illustrated in Figure 1.

### Gene mapping and normalization

**Figure 2:**
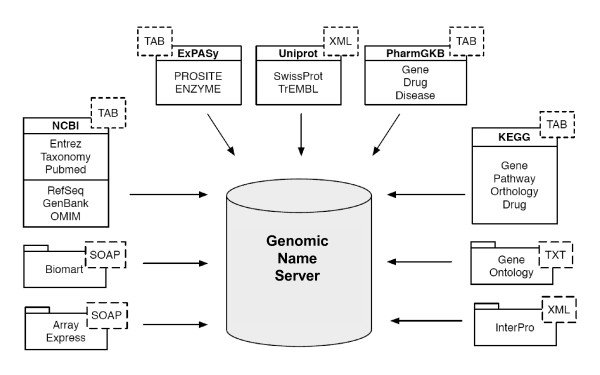
**Integration database schema**. Schematic representation of the databases integrated locally. The dotted boxes indicate the exchange format (XML, TAB, TXT) or method (SOAP) used to integrate the data.

The first problem is related to the different database identifiers and symbols used for the same gene across different databases and in the literature. This creates difficulties in data integration [26] and also when processing the user queries. To address these mapping issues, we developed a local database that integrates information from the most representative biological resources [27], including UniProt, Entrez Gene, KEGG and GO. For each database we selected the most adequate access method and developed a specific loader responsible for converting the data to a format compatible with our data integration schema. Figure 2 illustrates the selected databases and the methods used to extract the data. Altogether these databases represent over 150 different data types. By merging all of these data, we obtain over 7 million gene products and more than 140 million associations.

The term mapping scheme implemented in the database works as a thesaurus through which each gene identifier (or symbol) in the user input is validated. The input genes that are present in the database are mapped to an internal identifier which is then used in the following operations. This mapping is performed considering the organism selected by the user, therefore reducing the problem of homonymy between genes in different species.

### Query expansion

In terms of query expansion, we have followed two different perspectives. The first one deals with the different synonyms that can be found in the literature for a given gene [22-24]. For this, all know synonyms for each gene in the user input are obtained from the data integration database and included in the query.

The second perspective, concept-based query expansion [25,28], allows to obtain other documents that deal with concepts related to the input genes, like proteins or pathways. Our approach is based on exploiting the direct links between genes and other biological concepts obtained from public biological databases. These networks of associations are implemented through direct relations in the integration database mentioned above (see 'Gene mapping and normalization'). The following entries from this database are used currently: gene names and symbols (obtained from Entrez Gene and UniProt), protein names (from UniProt), pathway names (from KEGG) and diseases (from OMIM).

Query expansion is performed as follows: for each gene in the query, the algorithm obtains, from the term expansion table, all the alternative gene and protein names corresponding to that gene's ID, and the associated pathways and diseases. Since the internal identifier for each gene is used at this stage (see above), only the synonyms and concepts referring to the organism selected by the user are considered. The full list of terms from all input genes is then accumulated in four separate query strings (one for each concept type). Pathway and disease terms occurring for more than one gene in the input are given an increased weight, which is used in constructing the queries. These query strings are then used in document retrieval as described next. The reason for adding the term weights is that we want to give greater relevance to terms which are linked to two or more genes in the input. For example, if three genes in the user input have been linked to the same pathway or disease, abstracts containing that pathway or disease name will have their score increased.

### Document indexing and retrieval

For efficient document retrieval, QuExT uses a local index of the PubMed database, created with Lucene [29]. During indexing, both the document title and abstract are processed using the Lucene standard analyzer. These are tokenized and indexed as one single field, but not stored. Each indexed document is associated with the corresponding PubMed document identifier (PMID). This allows retrieving the corresponding document from PubMed once the query results are obtained, and eliminates the need to store the abstracts locally, therefore greatly reducing the storage requirements of the application.

Document retrieval is performed through Lucene. We run four index searches using the four query strings obtained in the query expansion stage (one for each concept type). For each search, we obtain the documents that match the query and the corresponding Lucene scores. These results are then organized in order to have, for each PubMed ID (PMID), a list of four scores: gene, protein, pathway and disease.

### Weighting scheme

In order to produce the final ranked list that is returned to the user, the results from the document retrieval stage are assembled and documents are re-ranked in terms of the defined weights for each concept type (see Figure 1). The final score for document *i *is obtained as a weighted sum of the four concept-based scores:(1)

where *W*_*j *_is the weight attributed to the concept type *j *and *s*_*ij *_represents the score for document *i *in terms of the *jth *concept type. This score is obtained from Lucene, as described above. In the example of Figure 1, the document with PMID 2142119 has a score of 5.00 for the concept type 'Genes', 3.47 for the concept type 'Proteins', and 3.00 for the concept type 'Diseases'. According to Equation 1, these scores are multiplied by the corresponding weight and added to produce the final score for this document (*5.0 × 0.5 + 3.47 × 0.3 + 0.0 × 0.1 + 3.0 × 0.1 = 3.84*). Scores are then normalized in terms of the highest one to obtain a relative score for each document, as shown in Figure 1.

This weighting scheme allows users to alter the order of the retrieved documents by simply changing the concept weights. This allows, for example, focusing on documents describing the metabolic pathways in which the initial genes are involved. From a user perspective, this is achieved by changing the value of the slide bars on the application's user interface. When the new weight values are set, the documents are re-ranked according to Equation 1, without the need to perform the query expansion or the document retrieval stages.

## Results and discussion

A wide range of web-based information retrieval tools offer optimized or specialized versions of the search functionalities provided by the PubMed database. Each of the available web-based systems has distinct features, and may be preferred by particular users or to address specific problems [7]. Despite that, the tool presented here offers three features that, to our knowledge, are not found in combination in any other tool. First, the automatic expansion of each gene identifier in the initial query to a set of other biologically related concepts. Second, the document retrieval and ranking method was designed to give higher relevance to terms that relate to more than one gene in the input list. This way, documents containing such terms will be ranked higher in the final result. Third, by allowing users to define different weights for the classes of concepts used in the query expansion, the tool allows users to explore the scientific literature from different perspectives, by focusing on particular aspects of the basic gene biology. Together, these characteristics make QuExT a unique tool for addressing the issue of selecting the most relevant papers that combine information relating to a set of genes, such as the outcome of a gene expression study.

### User interface

**Figure 3:**
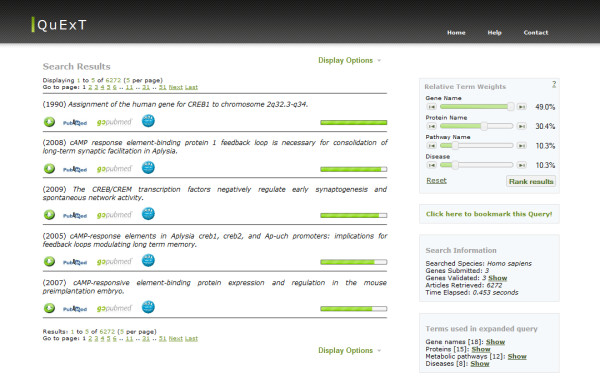
**QuExT user-interface: results explorer window**. QuExT user-interface. After submitting a list of genes, the returned documents are displayed in the results explorer window. The left panel shows the ranked documents; the right panel allows setting the weights of the concepts and shows information about the input genes and the terms used for query expansion. Users can see selected abstracts or open the corresponding citation in PubMed, GoPubMed or iHOP

The query expansion and document retrieval method described in this paper was implemented as a web-based application. The user interface is divided in two simple forms. The first one allows the user to insert a list of gene identifiers and select the organism of study. There is also the option to upload a text file with the list of genes. After submitting the query, the retrieved documents are presented in the results explorer interface (see Figure 3). The titles of the retrieved documents are displayed in the left panel, while the right-side panel shows the input genes and the gene-related concepts used in the expanded query. The user can expand each individual abstract or navigate to the corresponding entry in PubMed, GoPubMed or iHOP by using the corresponding button on the page. As explained above, the abstracts are not saved locally, but instead they are obtained from PubMed using the Entrez e-utilities [30] once the query results are returned. Also shown in Figure 3 are the slide bars used to change the concept weights used for ranking the documents. Setting the value of each slider changes the relative weight for each type of concept used for expanding the query. For example, setting the weight of the concept type 'Disease' to 100% (and the remaining to 0%, accordingly), will show the documents ranked in terms of their scores for this concept type only.

Four classes of concepts are currently used for query expansion, but the flexibility of the concept-based expansion and weighting scheme allows the inclusion of more concepts in a straightforward manner. For example, new concepts such as Biological Process terms (from the Gene Ontology), may be included to enrich the expansion. Another possibility is the inclusion of MeSH terms and resource identifiers for the concepts that appear in the document, such as UMLS concept IDs or UniProt accession numbers, in order to categorize the documents and offer links to the primary data sources describing each concept. Likewise, seven reference organisms are supported at present: Homo sapiens, Mus musculus, Rattus norvegicus, Candida albicans, Saccharomyces cerevisiae, Drosophila melanogaster and Apis mellifera. Inclusion of new organisms only requires a straightforward update of the information in the database and can be easily accomplished if there is demand by users.

### Retrieval performance

In order to assess the retrieval performance of the method, we performed an evaluation using data from the ad-hoc retrieval task in the 2004 TREC genomics track [31]. This task is based on the information needs of real biologists, and consists of 50 queries. However, since QuExT is a gene-centred application, not all queries could be used for performing this evaluation: general ones such as "Gene expression profiles for kidney in mice" or "Cause of scleroderma" had to be excluded since these cannot be addressed by QuExT. From the 50 available topics, 18 that were found as being more centred in a particular gene (or set of genes) were selected. Furthermore, since the approach in QuExT is to use as input a gene or a set of genes, rather than a more specified query such as the ones used in TREC, these queries had to be pre-processed to select just the gene names. For example, for the queries "Role of TGFB in angiogenesis in skin" and "Role of p63 and p73 in relation to DNA damage", only the gene name(s) are passed to QuExT ("TGFB" and "p63, p73", respectively). After this step, two queries that explore different roles of the same gene, namely "TGFB", became the same query for QuExT. These two queries, and the corresponding relevant documents, were therefore merged, producing a final set of 17 queries for the evaluation.

The evaluation test produced a mean average precision (MAP) of 0.425, with an average of 4.8 and 31.3 relevant documents within the top 10 and 100 retrieved abstracts, respectively. These results are comparable with the best systems in this task [31]. The best evaluation results were obtained with concept weights set at 100 for gene names/symbols, 10 for protein names and 0 for pathway names (the concept type "disease" was not used in this evaluation). Using pathway names produced a decrease in performance for this set of queries (MAP of 0.405 with pathways weight set at 5). Comparing to executing the search with no query expansion, we obtain an increase of 26% in the MAP statistic (from 0.337 when using just the gene names).

As another measure for comparison, we also ran the same evaluation using PubMed as the retrieval engine. For this, we used the Entrez e-utilities to obtain the first 10.000 documents for each gene query, limited to the date range in the 2004 TREC collection (from 1994 to 2003, inclusive). This experiment yielded a MAP value of 0.239, with 1.5 and 17.7 relevant documents within the top 10 and 100 retrieved abstracts, respectively.

These evaluation results indicate good overall results, especially when comparing to the use of PubMed. Although PubMed uses query expansion through Automatic Term Mapping (ATM), and includes manually annotated information in the search, through MeSH terms [32], our evaluation results show a 78% increase in the MAP statistic when using QuExT (0.425), as compared to PubMed (0.239). The reduced performance obtained for this task with PubMed (as measured by MAP) is related to the lack of any relevance ranking of the resulting documents, leading to relevant documents appearing lower in the returned list.

### Results analysis

Although performance measures based on a manually annotated test set gives some indication of the method's retrieval performance, the 2004 TREC genomics evaluation data and methodology may not be entirely suited for testing our method. First of all, QuExT is oriented to a very specific query type. In contrary, the systems used in a task such as the ad-hoc retrieval in TREC are usually more generic IR tools that can be tuned to match the requirements of the task. Another limitation, which is related to the later, is the fact that not all query types in the TREC task could be used to evaluate QuExT. Since this is a gene-centred application, we had to exclude the majority of the queries, ending up with 17, which were considered to be more focused on a particular gene (or set of genes). These limitations imply that the results obtained do not allow a completely valid comparison with other systems that use the same evaluation, including the ones that participated in the TREC task and other more recent ones. Also, even if the same evaluation set could be used, it is not straightforward to compare QuExT to these tools, as the philosophy and approaches are very dissimilar. Finally, although our evaluation tests with the TREC data show that the inclusion of gene-related concepts helps improving retrieval performance and that optimal results can be obtained with different weight settings, this latter feature is still the most difficult to evaluate in an objective manner. It is important to evaluate this feature from the point of view of users, since it gives them a simple way to influence the ranking of the results in order to obtain the documents that better reflect their information need. In order to do so, we have asked a group of researchers to use the tool and compare it to the use of PubMed. The initial observations obtained indicate that this tool, and specially the concept of using weights to modify the relevance of the results, may be very useful to researchers studying a set of genes. Although this small study gave some good indications about the validity of the approach, the results are very subjective and a more structured evaluation, using a set of well-defined and validated information requests, is required and will be the subject of future research.

### Future work

An important difficulty in information retrieval systems in biomedicine has to do with matching entity names, such as gene, protein and pathway names, to their occurrence in the texts. At this stage, we rely upon the use of well established terminological resources extracted from reference biomedical databases. Namely, we use the Entrez Gene database for gene names and symbols, UniProt for protein names, KEGG for pathway names, and the OMIM database for disease names. However, these resources do not cover all possible textual variations for a given term. For instance the gene "ATP-binding cassette sub-family A member 1" can appear in different ways in the literature. This includes lexical variation from the full gene name (e.g. word suppression or different word order) as well as morphological variations in gene symbols (ABC1, ABC-1 or ABC 1). Our indexing method uses the standard document analyzer and tokenizer implementations in Lucene, and does not deal with these variations entirely. Our ongoing research includes investigating the use of string similarity algorithms and rule-based strategies during indexing time in order to deal with this limitation and improve the retrieval performance. Namely, we are working on specific tokenization rules to normalize gene/protein symbols to a canonical form in order to address these morphological variations. In this regard, techniques similar to the methods that have been proposed in the literature [22,23] are being considered.

Another major difficulty is ambiguity, that is, the same symbol identifying different genes and/or proteins. In QuExT, this problem is reduced when performing the mapping and query expansion, since the user is requested to select an organism for the input genes. Furthermore, the inclusion of related terms in the query also addresses this problem, as documents containing more terms associated to the input genes will have a higher ranking. For example, an abstract containing the terms 'CAT' and 'catalase' will have higher relevance than an abstract containing just the term 'CAT'. This aspect can be further explored by introducing reliability (or ambiguity) scores to the terms in our thesaurus.

## Conclusions

This paper presents QuExT, a document retrieval and prioritization tool specially designed to obtain from the literature the most relevant articles relating to a given list of genes. QuExT follows a concept-oriented query expansion methodology to find documents containing concepts related to the genes in the user input, such as protein and pathway names. The main innovation of the proposed application is the possibility to modify the weights for the four concept classes used in the query expansion: gene names and symbols, protein names, metabolic pathways and diseases. This gives the user control on how the expanded search terms affect the final ranking of the documents. The user can, for example, select to give more strength to the pathway names in the search query, therefore bringing to the top of the results those documents in which pathway names relevant to the initial gene list appear. Although a more detailed evaluation and comparison with other tools is required, initial evaluation based on data from the TREC genomics track indicates good retrieval performance. The application of concept weights to modify the order of the returned documents represents a significant advantage when compared with the available methods.

## Availability and requirements

Project name: QuExT - Query Expansion Tool

Project home page: http://bioinformatics.ua.pt/quext

Operating system(s): Platform independent

Programming language: Python

Other requirements: n/a

License: n/a

Any restrictions to use by non-academics: no restrictions

## Authors' contributions

SM, JPA and JMR participated in the design and implementation of the system and drafted the manuscript. JLO conceived the study, and participated in its design and coordination and helped to draft the manuscript. All authors read and approved the final manuscript.

## References

[B1] Altman R Bergman C Blake J Blaschke C Cohen A Gannon F Grivell L Hahn U Hersh W Hirschman L Jensen LJ Krallinger M Mons B O'Donoghue SI Peitsch MC Rebholz-Schuhmann D Shatkay H Valencia A Text mining for biology - the way forward: opinions from leading scientists Genome Biol 2008 9 Suppl 2 S7 10.1186/gb-2008-9-s2-s7 18834498 PMC2559991

[B2] Jensen LJ Saric J Bork P Literature mining for the biologist: from information retrieval to biological discovery Nat Rev Genet 2006 7 2 119 129 10.1038/nrg1768 16418747

[B3] Rebholz-Schuhmann D Kirsch H Couto F Facts from text-is text mining ready to deliver? PLoS Biol 2005 3 2 e65 10.1371/journal.pbio.0030065 15719064 PMC548955

[B4] Shatkay H Hairpins in bookstacks: information retrieval from biomedical text Brief Bioinform 2005 6 3 222 238 10.1093/bib/6.3.222 16212771

[B5] Krallinger M Valencia A Text-mining and information-retrieval services for molecular biology Genome Biol 2005 6 7 224 10.1186/gb-2005-6-7-224 15998455 PMC1175978

[B6] Manning C Raghavan P Schütze H Introduction to Information Retrieval 2008 New York: Cambridge University Press

[B7] Kim JJ Rebholz-Schuhmann D Categorization of services for seeking information in biomedical literature: a typology for improvement of practice Brief Bioinform 2008 9 6 452 465 10.1093/bib/bbn032 18660511

[B8] Weeber M Kors JA Mons B Online tools to support literature-based discovery in the life sciences Brief Bioinform 2005 6 3 277 286 10.1093/bib/6.3.277 16212775

[B9] Doms A Schroeder M GoPubMed: exploring PubMed with the Gene Ontology Nucleic Acids Res 2005 33 Web Server W783 786 10.1093/nar/gki470 15980585 PMC1160231

[B10] Hoffmann R Valencia A A gene network for navigating the literature Nat Genet 2004 36 7 664 10.1038/ng0704-664 15226743

[B11] Plake C Schiemann T Pankalla M Hakenberg J Leser U AliBaba: PubMed as a graph Bioinformatics 2006 22 19 2444 2445 10.1093/bioinformatics/btl408 16870931

[B12] Rebholz-Schuhmann D Kirsch H Arregui M Gaudan S Riethoven M Stoehr P EBIMed-text crunching to gather facts for proteins from Medline Bioinformatics 2007 23 2 e237 244 10.1093/bioinformatics/btl302 17237098

[B13] Tsuruoka Y Tsujii J Ananiadou S FACTA: a text search engine for finding associated biomedical concepts Bioinformatics 2008 24 21 2559 2560 10.1093/bioinformatics/btn469 18772154 PMC2572701

[B14] Chen H Sharp BM Content-rich biological network constructed by mining PubMed abstracts BMC Bioinformatics 2004 5 147 10.1186/1471-2105-5-147 15473905 PMC528731

[B15] Miyao Y Ohta T Masuda K Tsuruoka Y Yoshida K Ninomiya T Tsujii J Semantic retrieval for the accurate identification of relational concepts in massive textbases Proceedings of the 21st International Conference on Computational Linguistics and 44th Annual Meeting of the Association for Computational Linguistics: 17-21 July 2006 2006 Sydney, Australia. Association for Computational Linguistics 1017 1024

[B16] Arrais J Santos B Fernandes J Carreto L Santos MAS Oliveira JL GeneBrowser: an approach for integration and functional classification of genomic data J Integr Bioinform 2007 4 3

[B17] Draghici S Khatri P Martins RP Ostermeier GC Krawetz SA Global functional profiling of gene expression Genomics 2003 81 2 98 104 10.1016/S0888-7543(02)00021-6 12620386

[B18] Korotkiy M Middelburg R Dekker H van Harmelen F Lankelma J A tool for gene expression based PubMed search through combining data sources Bioinformatics 2004 20 12 1980 1982 10.1093/bioinformatics/bth183 15044238

[B19] Schuemie MJ Kang N Hekkelman ML Kors JA GeneE: gene and protein query expansion with disambiguation Bioinformatics 2010 26 1 147 148 10.1093/bioinformatics/btp597 19837720

[B20] Arrais J Rodrigues J Oliveira J Corchado JM, De Paz JF, Rocha MP, Fernández-Riverola F Improving Literature Searches in Gene Expression Studies Proceedings of the 2nd International Workshop on Practical Applications of Computational Biology and Bioinformatics (IWPACBB 2008): 22-24 October 2008; Salamanca, Spain 2009 Berlin: Springer 74 82 full_text

[B21] Chen L Liu H Friedman C Gene name ambiguity of eukaryotic nomenclatures Bioinformatics 2005 21 2 248 256 10.1093/bioinformatics/bth496 15333458

[B22] Schuemie MJ Mons B Weeber M Kors JA Evaluation of techniques for increasing recall in a dictionary approach to gene and protein name identification J Biomed Inform 2007 40 3 316 324 10.1016/j.jbi.2006.09.002 17079192

[B23] Koike A Takagi T Gene/protein/family name recognition in biomedical literature Proceedings of BioLINK 2004: linking biological literature, ontologies, and databases: 6 May 2004; Boston 2004 Association for Computational Linguistics 9 16

[B24] Lu Y Fang H Zhai C An empirical study of gene synonym query expansion in biomedical information retrieval Inf Retr 2009 12 1 51 68 10.1007/s10791-008-9075-7

[B25] Stokes N Li Y Cavedon L Zobel J Exploring criteria for successful query expansion in the genomic domain Inf Retr 2009 12 1 17 50 10.1007/s10791-008-9073-9

[B26] Pinto J Dias O Lourenço A Carneiro S Ferreira E Rocha I Rocha M Corchado JM, De Paz JF, Rocha MP, Fernández-Riverola F Data Integration Issues in the Reconstruction of the Genome-Scale Metabolic Model of Zymomonas Mobillis Proceedings of the 2nd International Workshop on Practical Applications of Computational Biology and Bioinformatics (IWPACBB 2008): 22-24 October 2008; Salamanca, Spain 2009 Berlin: Springer 92 101 full_text

[B27] Arrais J Pereira JE Fernandes J Oliveira JL GeNS: a biological data integration platform Proceedings of the International Conference on Bioinformatics and Biomedicine (ICBB 2009): 26-29 October 2009; Venice, Italy 2009 850 855

[B28] Qiu Y Frei H-P Concept based query expansion Proceedings of the 16th Annual International ACM SIGIR Conference on Research and Development in Information Retrieval: 27 June - 1 July 1993; Pittsburgh, PA 1993 ACM 160 170 full_text

[B29] Apache Lucene http://lucene.apache.org/

[B30] Entrez Programming Utilities http://eutils.ncbi.nlm.nih.gov/corehtml/query/static/eutils_help.html

[B31] Hersh WR Bhupatiraju RT Ross L Roberts P Cohen AM Kraemer DF Enhancing access to the Bibliome: the TREC 2004 Genomics Track J Biomed Discov Collab 2006 13 1 3 10.1186/1747-5333-1-3 PMC1440302 16722581

[B32] Lu Z Kim W Wilbur WJ Evaluation of Query Expansion Using MeSH in PubMed Inf Retr 2009 12 1 69 80 10.1007/s10791-008-9074-8 PMC2747526 19774223

